# Spatial scales, patterns, and positivity trends of SARS-CoV-2 pandemics in mass rapid antigen testing in Slovakia

**DOI:** 10.1371/journal.pone.0256669

**Published:** 2021-08-25

**Authors:** Katarína Boďová, Richard Kollár

**Affiliations:** Faculty of Mathematics, Physics, and Informatics, Comenius University, Bratislava, Slovakia; Waseda University: Waseda Daigaku, JAPAN

## Abstract

We study geographical epidemic scales and patterns and positivity trends of SARS-CoV-2 pandemics in mass antigen testing in Slovakia in 2020. The observed test positivity was exponentially distributed with a long scale exponential spatial trend, and its characteristic correlation length was approximately 10 km. Spatial scales also play an important role in test positivity reduction between two consecutive testing rounds. While test positivity decreased in all counties, it increased in individual municipalities with low test positivity in the earlier testing round in a way statistically different from a mean-reversion process. Also, non-residents testing influences the mass testing results as test positivity of non-residents was higher than of residents when testing was offered only in municipalities with the highest positivity in previous rounds. Our results provide direct guidance for pandemic geographical data surveillance and epidemic response management.

## Introduction

From the earliest stages of the SARS-CoV-2 pandemic in spring 2020 various non-pharmaceutical interventions were enforced in hope to control the local epidemic situation. Mass testing with antigen tests that identify the presence of viral proteins expressed by the virus [1–3] was a common choice in Europe in autumn 2020 due to the point-of-care test application and fast result delivery [4–6]. As any mitigation measure, in addition to its advantages, mass antigen testing has its own limitations and costs [7–14].

**Fig 1:**
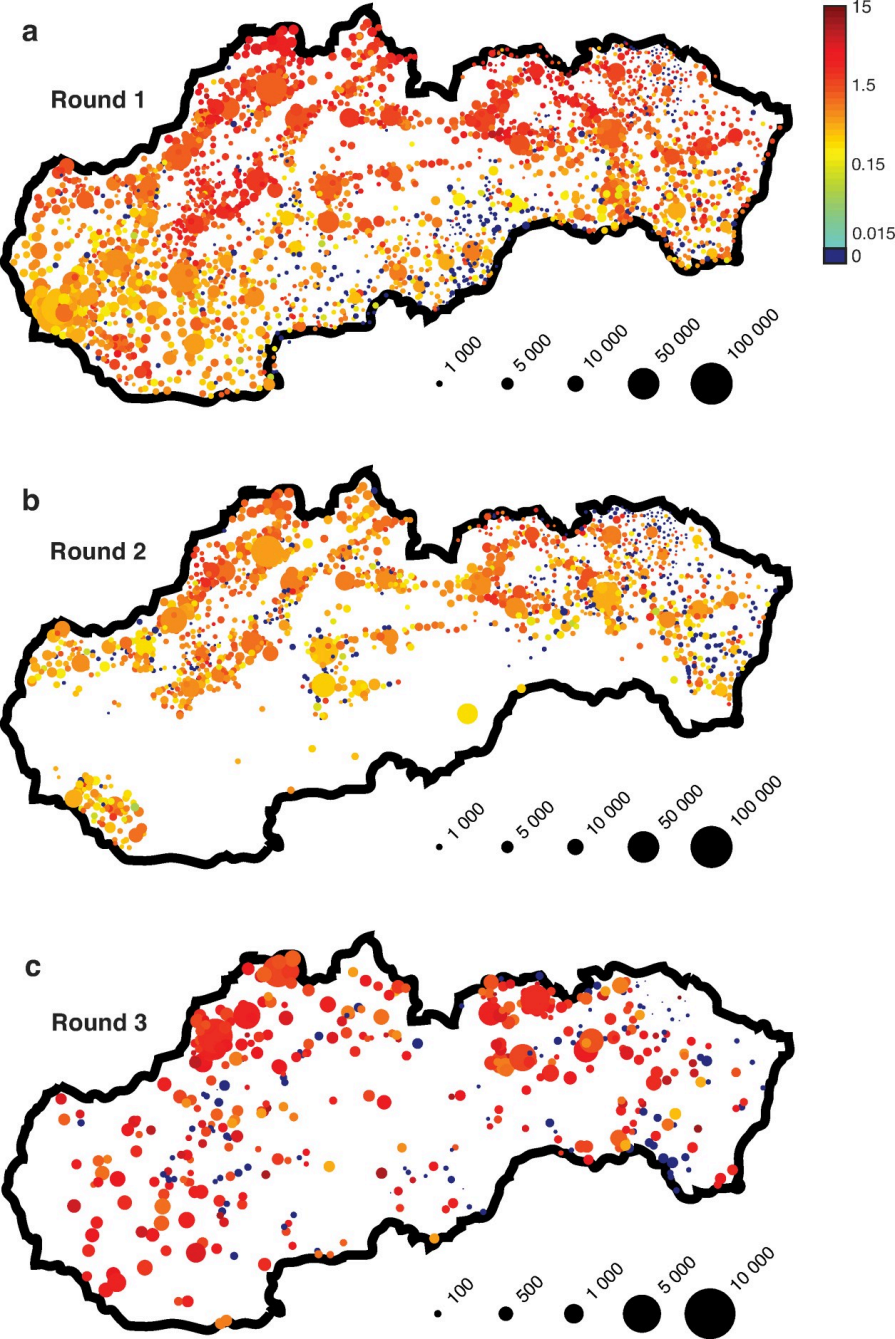
**a-c** Results of mass antigen testing in Rounds 1–3. Test positivity (%) for individual municipalities is encoded by a logarithmic color scale. Marker size indicates the total number of tests administered. Due to a lower number of tests in Round 3, marker size is proportionally increased for better data visibility. See S1 Code and dataset for data and code used to generate the figures.

The Slovak Republic (population 5.45 millions, area 49,035 km^2^) conducted mass antigen testing in October and November 2020 as the first SARS-CoV-2 associated large scale mass testing in Europe [15–18]. The mass testing campaign in Slovakia consisted of the pilot round (Round 0) and three regular testing rounds (Rounds 1–3), see Table 1 and Fig 1. Round 0 was limited to four counties with the highest current 7-day incidence of positive real time quantitative polymerase chain reaction tests (RT-qPCR) with three of the counties located near the large observed geographical SARS-CoV-2 epidemic cluster in Poland and Czech Republic [19]. Round 1 (one week later) comprehensively covered the whole country, Round 2 (one week after Round 1) was limited to the counties with high Round 1 test positivity and Round 3 (two weeks after Round 2) was limited to municipalities with high test positivity in Rounds 1–2. The mass testing was combined with additional mitigation measures to limit mobility [13–18], see Appendix B in S1 Text. Untested residents of the counties where testing was administered were subject to strict mobility restrictions including mandatory quarantine for 7–14 days. In Round 3 testing was voluntary and no mobility restrictions in addition to general mitigation measures were imposed to untested individuals.

**Table 1:** Summary of the mass testing in Slovakia (total 79 counties).

	Date	TestingStations	Number of Counties	Number of Municipalities	Number of tests	Number of positive tests	Test positivity
**Pilot**	Oct 23–25	235	4	--	140,945	5,594	3.97%
**Rd 1**	Oct 31-Nov 1	4782	79	2693	3,625,332	38,359	1.06%
**Rd 2**	Nov 7–8	2756	45	1580	2,044,885	13,509	0.66%
**Rd 3**	Nov 21–22	--	--	447	110,609	2,501	2.26%

The observed mass antigen test positivity (further referred to as “positivity”) provided a systematic measure of the local epidemic situation as more than 60% of the population were tested under similar conditions over a short period of time. However, positivity cannot be directly related to the prevalence due to systematic biases in the tested samples [13].

Despite these interpretation limitations positivity provides a unique source of information and the mass antigen testing in Slovakia is a source of the first publicly available dataset that comprehensively characterizes the local epidemiological situation during the SARS-CoV-2 pandemics on the level of individual municipalities across a whole European country. The data allow us to study emerging geographical scales, patterns and trends, see [20–23] for studies of geographical spreading and patterns of SARS-CoV-2 pandemic using other data sets.

## Methods

### Data

Our analysis is based on the existing publicly available aggregated data published by the Department of Defense of the Slovak Republic and by the Institute for Healthcare Analyses of the Department of Health of the Slovak Republic [24, 25]. Round 0 results were reported on county level only. Round 1–3 results (total number of tests and positive tests) were reported on the level of individual municipalities and city districts. In cities with reporting from multiple districts we combined the data into city totals (Bratislava 5 districts, Košice 4 districts). In Round 3 the data were reported separately for residents and non-residents in each municipality. For geographical data analysis we obtained GPS coordinates of each municipality in the data set from National Geospatial-Intelligence Agency [26].

### Tests

SD Biosensor Standard Q Covid Ag (SD Biosensor, Inc., Gyeonggi-do, Korea) kit [27], also distributed by Roche [28] was used in all administered tests. The manufacturer claimed sensitivity 96.52% (95% CI 91.33–99.04%) and specificity 99.68% (95% CI 98.22–99.99%) in the kit leaflet [27] at the time of testing. See Appendix A in S1 Text for a survey of test validation studies with sensitivity median 71.6% and average 72.1%, and specificity median 99.3% and average 98.6%. The lower bound for test specificity in mass testing in Slovakia was estimated at 99.6% in [29]. Mass testing methodology and setup are described in detail in [14, 15], see also Appendix B in S1 Text.

### Data analysis

We analyzed the data using three methods.

Nonlinear regression identifies relationships within the dataset and assesses their significance level. We used the standard notation for the p-values: *, **, *** mean p-value less than 0.1, 0.01, and 0.001, respectively. Data were fitted with built-in tools for nonlinear regression (nlinfit and nlparci) and a Curve Fitting Toolbox for weighted linear regression in MATLAB©. The data and the codes generating the figures can be found in S1 Code and dataset.

Global Moran’s *I* measures spatial autocorrelation in discrete geographical data. Its value for data {*x*_*i*_} in regions with index *i* ∈ {1, …, n} is given by

$$I=\frac{n\sum_{i=1}^{n}\sum_{j=1}^{n}w_{ij}(x_{i}-X)(x_{j}-X)}{S_{0}\sum_{i=1}^{n}\left(x_{i}-X\right)},$$

and it is computed for a particular choice of spatial weights *w*_*ij*_ that encode contiguity of regions with indexes *i* and *j*. Here *X* is the data average in all regions, and S_0_ is a total sum the spatial weights w_ij_:

$$S_{0}=\sum_{i=1}^{n}\sum_{j=1}^{n}w_{ij}.$$

Global Moran’s *I* is frequently used for a statistical analysis of geographical patterns, e.g. in spatial studies of SARS-CoV-2 pandemics in China [20–23].

We computed Global Moran’s *I* for antigen test positivity. To identify the characteristic spatial correlation length in the data we tiled the area of Slovakia by the same-size square regions, computed the average test positivity in each region, and computed the Global Moran’s *I*. The characteristic correlation length was obtained by a maximization of the value of the statistics for tilings with square sides 3, 4, …, 50 km.

**Fig 2:**
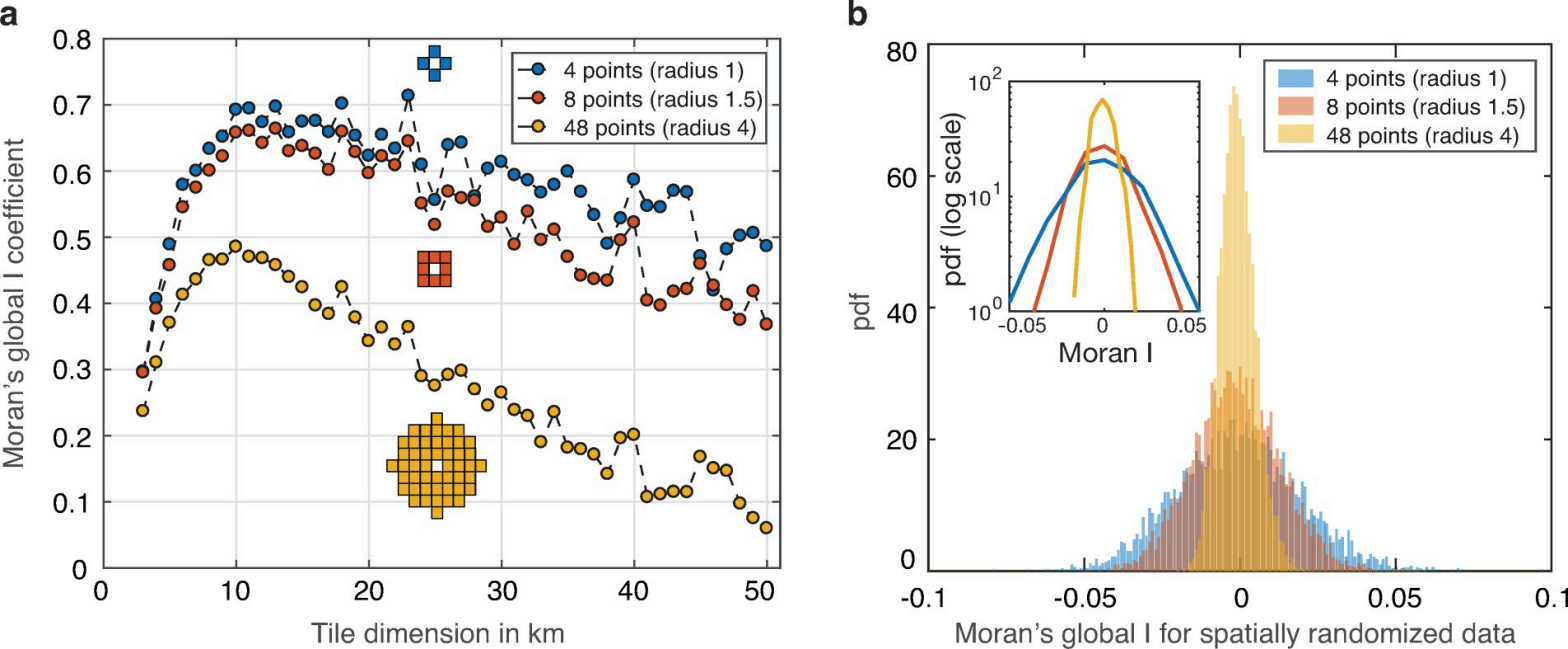
Spatial correlation of Round 1 positivity. **a** Moran’s I statistics plotted against the dimension of the square tiles covering the area of Slovakia. For each tile dimension Moran’s I values for three alternative weight functions are displayed, see Methods. **b** Distribution of Moran’s I for randomly reshuffled data (4999 permutations) for the three weight functions and tile dimension 10 km. The distributions on a logarithmic scale are shown in the inset.

As Global Moran’s *I* is sensitive to the choice of the definition of spatial regions contiguity, we considered three alternative tile contiguity definitions for each tile dimension: two tiles are contiguous if their centers are no more than 1-, 1.5-, and 4-times the tile dimension apart. Thus each tile had at most 4, 8, and 48 neighbors, respectively (see the diagrams in Fig 2A), as only the tiles overlapping with the area of the Slovak Republic were included in our analysis. In all our contiguity definitions all pairs of contiguous tiles contributed equally to the Moran’s I statistics weight function and non-contiguous tile pairs had zero weight.

To quantitatively detect and analyze exponential geographical patterns in Round 1 test positivity we used radial spatial averaging of the data from various focal locations. High level of local data variation, multiple geographical sources, and high irregularity in spatial data locations blur the local analysis and robust identification and deconvolution of spatial exponential trends. Therefore we limited our search for exponential spatial patterns to large scales comparable with the size of the country.

Our method was based on the observation that a strong exponentially localized source of a data signal in two dimensions is also approximately exponentially localized when observed from a distant location (focal point) through radial averaging over annular regions of an increasing radius and of the same width. Thus radial averaging of the data from a focal point allows to identify dominant exponential spatial patterns in the studied data.

**Fig 3:**
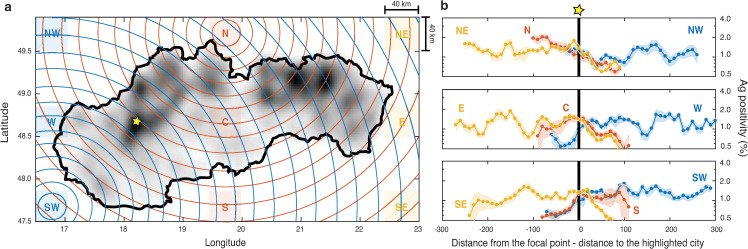
Geographical distribution of positivity. **a** Round 1 positivity (increasing light to dark gray). For visualization purposes positivity was smoothened by diffusion (diffusion coefficient 0.05 km^2^ /s, final time 0.45 s) to average out a local variation. 9 focal areas are indicated as shaded rectangles. **b** Dependence of average positivity (log scale) on the distance from 9 focal areas; shaded values at each distance correspond to mean±std across the 247 focal points in the focal area. Distance from each grid point in the averaged ensemble in each focal area is normalized so that the reference location of high positivity (indicated by a yellow star in **a**) is shifted to the origin on the horizontal axis (marked by a black line). See Methods for more details.

To eliminate high local variation in the considered data we first averaged the test positivity over a regular rectangular grid (approximately 2.3 x 3.3 km) covering the area of the Slovak Republic (Fig 3A). Local positivity in each grid rectangle was calculated as positivity of all tests administered in the municipalities centered within the rectangle.

Next, we calculated the average radial Round 1 test positivity from centers of selected grid rectangles. To eliminate sensitivity and eventual bias introduced by an exact selection of a focal point we have introduced larger focal areas of size approximately 40 km x 40 km. In each of them we further averaged the annular averages calculated for 247 focal points located within the focal area. In the ensemble average in each focal area we normalized the data from individual focal points so that the reference location of high positivity (indicated by a yellow star in Fig 3A) was shifted to the same distance from the focal point (denoted as 0). The resulting data for each focal area then represented an average test positivity at a given average distance from the focal area.

We considered eight focal areas located in the cardinal and intercardinal directions (denoted as N, NE, E, SE, S, SW, W, NW, W) and a central focal area (C), see Fig 3A. Note that eight of these focal areas lie outside of the area Slovak Republic, i.e., the resulting averaged radial data represent the view of the geographical test positivity patterns in Slovakia as seen from a location abroad. Examples of the annular regions, corresponding to the focal location in the centers of the regions N and SW, are shown in Fig 3A (with boundaries marked as red and blue concentric circles).

## Results

### Characteristic spatial scales and patterns in test positivity

The data from comprehensive Round 1 mass antigen testing in Slovakia reveal that

1. the characteristic spatial scale of positivity was approximately 10 km,
2. there were long scale exponential spatial trends in positivity that decreased from north to south, a homogenization effect was seen at large distances in the main axial direction of the orientation of the Slovak Republic.

(1) The characteristic spatial scale of test positivity in Round 1 was approximately 10 km. For this distance the maximum of the global Moran’s *I* statistics of spatial autocorrelation was robustly reached for all three considered definitions of contiguity of the regions, see Fig 2A. At short spatial scales (< 5 km) low positive spatial autocorrelation was observed, reflecting high variability in test positivity between individual (mostly small) municipalities. At long spatial scales (> 20 km) a systematic decay of spatial correlation was observed reflecting a low level of interconnection between more distant regions. The choice of one of the three weight functions that characterize which pairs of the regions are considered contiguous influenced only an overall magnitude of the Moran’s *I* statistics but the peaks of the statistics were located approximately at the same characteristic length.

To confirm that the values of the computed global Moran’s *I* at the distance 10 km (0.69, 0.66, 0.49 for three choices of a contiguity definition) are statistically significant we compared them with the values for random spatial assignment of data using a permutation test. In this test we compared the value of the *I* statistics for the real data with a distribution of the *I* values for 4999 random geographical permutations of the data values. Fig 2B shows that for all three considered definitions of contiguity Moran’s *I* for a random permutation of the data has an underlying distribution with a mean close to 0. Note that the expected value of Moran’s *I* for data with no spatial autocorrelation is *-1/(n-1)* where *n* is the number of regions considered. Each distribution (shown also in the semilogarithmic plot in the inset) is very narrow compared to the values of the Moran’s *I* for the real data. Thus the results of the permutation test imply that a test positivity permutation removes its spatial correlation and that at the significance level with the p-value < 0.001 the Moran’s *I* for Round 1 test positivity is significantly positive at a characteristic length 10 km, irrespective of the choice of the contiguity definition.

(2) Geographical patterns in Round 1 positivity are demonstrated by spatial gradients displayed in Fig 3A. The location of the highest observed positivity is indicated by a yellow star. The local trends around individual positivity peaks are blurred by a convolution of signals from multiple sources of high positivity.

On a long spatial scale comparable with the size of the whole country the convolution effects can be eliminated by radial spatial averaging of positivity observed from distant locations (see Methods). We calculated the radial spatial averages of Round 1 positivity from nine focal areas: N, NE, E, SE, S, SW, W, NW, W, and C (Fig 3B). The focal areas were located near the map edges in the cardinal and the intercardinal directions (outside of the area of Slovakia) and at the map center. The results show multiple long scale exponential trends (corresponding to linear trends on log-linear scale) in positivity. Foremost, the radial spatial positivity average from the focal area N decreased approximately exponentially with the distance from the focal area across all distances. The trend is consistent with the radial averages from NE and NW focal areas around the location of high positivity indicated by the star. Similar inverse trends in the opposite direction were observed from the opposite focal areas S and SW. However, the radial spatial average from the focal area SE is different as the radial averages at large distances from SE focal area average out regions of high and low positivity in the north and south of the western part of Slovakia. A homogenization effect (constant positivity trend) is seen at large distances from the focal areas SW and NE in the main axial direction of the orientation of the Slovak Republic.

### Trends in test positivity

The geographical data from Rounds 1–3 of the mass antigen testing in Slovakia reveal important trends and effects:

3 positivity in individual municipalities was exponentially distributed,
4 positivity of non-residents was higher than positivity of residents in Round 3,
5 the extent of reduction of positivity in two consecutive testing rounds increased with the test positivity in the earlier round,
6 positivity increased between two consecutive rounds of mass testing in municipalities with very low positivity in the earlier round in a statistically different manner from a simple mean reversion process,
7 the correlation between the test results in two consecutive rounds increased when considered on larger spatial scale (counties vs. municipalities),
8 mass antigen test results were positively correlated with the recent 7-day RT-qPCR incidence in the first round of testing but a significant correlation disappeared in the next round of testing.

**Fig 4:**
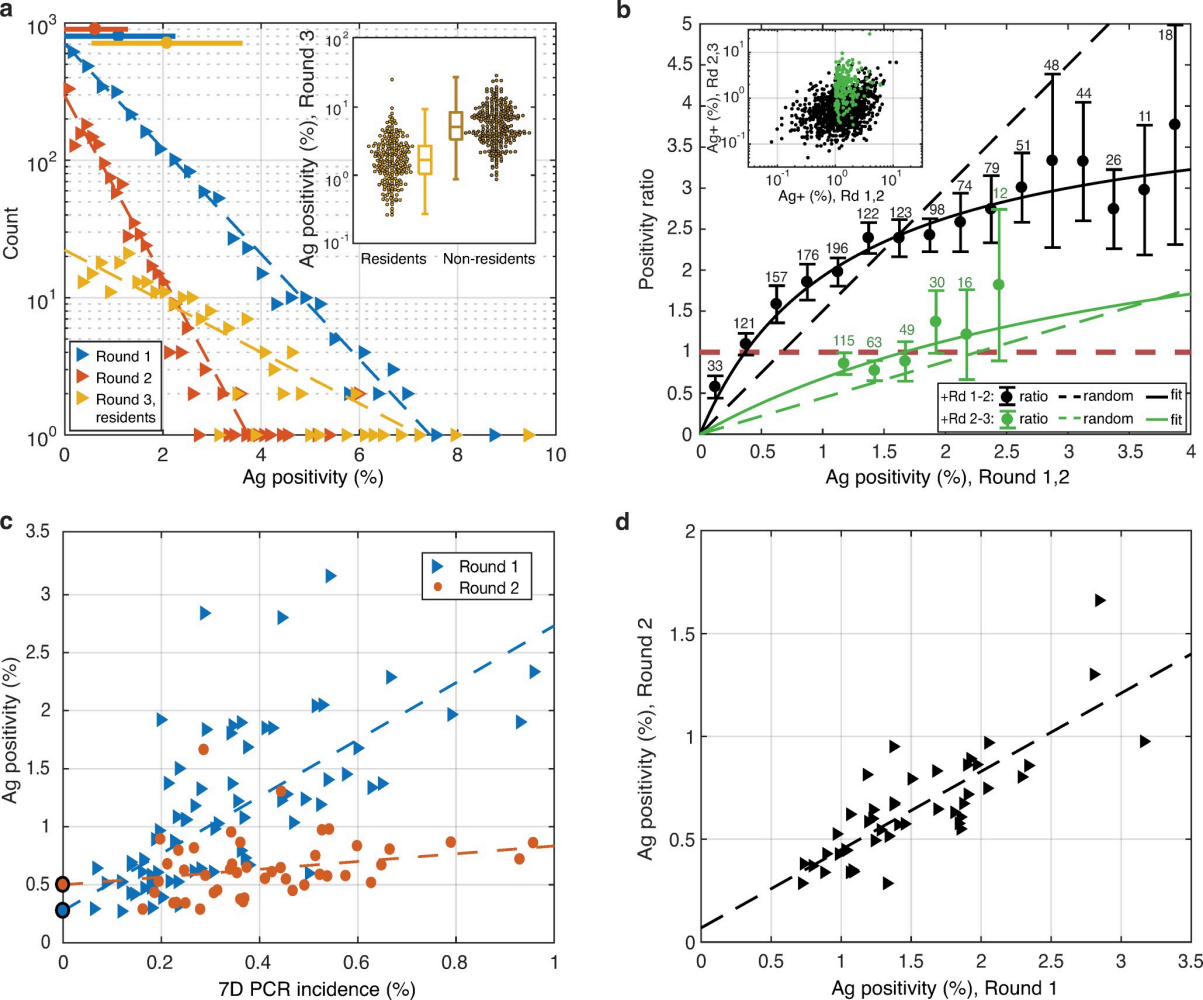
Graphical statistical data analysis. **a** Histograms of positivity in municipalities in Rounds 1–3. Each triangle represents a number of municipalities within the corresponding positivity bin. Horizontal color bars on the top indicate mean±std. Dashed lines correspond to exponential fits (S1 Table). Inset shows distributions of positivity for residents (n = 233) vs. non-residents (n = 265) in Round 3 (boxplots included). Zero data is not displayed. **b** Ratio of positivity in Round 2 vs. Round 1 and Round 3 (residents) vs. Round 2. Municipality based Ag positivity in the subsequent rounds is shown in the inset (only positive data displayed). The vertical bars correspond to 95% confidence intervals obtained by jackknife subsampling of the municipalities within the bin (withholding 50% of the data) with repetition. **c** Positivity in Rounds 1 and 2 against 7-day PCR incidence prior to Round 1. Dashed lines correspond to linear fits weighted by the population size. **d** Positivity in Round 2 vs. Round 1 for counties where both testing rounds were administered (n = 45) and the weighted linear fit (S1 Table).

Fig 4 shows a graphical statistical summary of the results. Also see S1 Table for parameters of all linear weighted and unweighted fits shown in Fig 4.

(3–4) Positivity in individual municipalities in Rounds 1–2 was well approximated by an exponential distribution (see Fig 4A). The exponential distribution was not observed for all tests in Round 3 (not shown) as the results were skewed by high participation of non-residents (22.6% of all test samples) and their relative high average positivity (3.88%, compared to average residents positivity 1.13%). However, when only residents were considered, Round 3 test positivity was also well approximated by an exponential distribution. See the inset of the Fig 4A for the graphical comparison of positivity distribution of residents and non-residents with nonzero positivity in Round 3 in individual municipalities.

(5) The test positivity in an earlier round of the mass testing influenced the positivity reduction from the earlier round to the next round. We compared the ratio of means of positivity (positivity ratio) in the earlier and in the later of the two consecutive rounds (Rounds 1–2 and Rounds 2–3) with the positivity in the earlier of the two compared rounds (Fig 4B, main panel). To eliminate the effect of testing non-residents we used only residents data in Round 3. Data in the earlier of the two compared rounds were binned to smooth out an individual positivity variation (all bins have width 0.25%, the number of municipalities in each bin is reported in the plot). The positivity ratio less than one, respectively larger than one, corresponds to an increase and a decrease in test positivity, respectively.

The ratio between Rounds 1 and 2 systematically increased with the positivity in Round 1 for the bins up to 3%, and it is approximately twice as large as the positivity ratio in Rounds 2 and 3 indicating larger positivity reduction in Round 2 than in Round 3. Moreover, the positivity ratio was close to 1 between Rounds 2 and 3 for bins with Round 2 positivity <1.5% and it did not change significantly for the bins with positivity between 1% and 2%.

(6) Rounds 1 and 2 positivity ratio was less than 1 when the Round 1 positivity was less than 0.5% indicating an increase of positivity in Round 2 compared to Round 1.

At a first glance the observed trend in Fig 4B can be explained by a mean reversion process: low positivity in the earlier round resulted (on average) in an increase in positivity in the next round while high positivity resulted (on average) in a decrease. To test whether the observed trend can be explained by mean reversion we compare two scenarios. First we model the mean reversion (dashed lines). We independently randomly shuffle test positivity in individual municipalities and observe a linear increase of the positivity reduction on the positivity in the earlier round. However, this dependence is not in agreement with the observed data. Instead, we used a linear fit of the positivities in two consecutive rounds (see Fig 4B inset and S1 Table for parameter values) and applied the linear fit to a calculation of the positivity reduction (solid curves). Unlike mean reversion this model captures the data tightly with the error bars containing the predicted value for all cases shown, suggesting that the increase in the reduction of the test positivity between two consecutive rounds of the mass testing (Rounds 1 and 2) cannot be explained purely by a mean reversion process but shows a better agreement with the assumption that test positivity reduction linearly increased with the test positivity in the earlier round.

(7) A comparison of positivity in Rounds 1 and 2 is displayed in Fig 4B inset, note the double logarithmic scale. See S1 Table for the linear fit parameters used to compute the solid lines in Fig 4B. Correlation in test positivity is higher (R-squared = 0.23) if data were weighted by the total volume of tests in the earlier of the two compared rounds then for unweighted data (R-squared = 0.10). Positivities in Rounds 2 and 3 appear to be uncorrelated (R-squared = 0.02 unweighted, 0.008 unweighted). Significantly stronger correlation (R-squared = 0.46) is observed on the counties level (Fig 4D main panel) suggesting a key role of a spatial scale considered.

(8) The relationship between antigen tests positivity and RT-qPCR test incidence in all counties is displayed in Fig 4C. Antigen test positivity in Round 1 is compared with the 7-day incidence of positive RT-qPCR tests per one county resident one day prior to the start of Round 1 (Oct 24–30). Unavailability of testing stations, low numbers of indications for testing by Regional Public Health Authorities, etc. caused disruptions in RT-qPCR data after mass testing commenced. Round 2 mass testing data are thus compared with the RT-qPCR incidence data delayed by 7 days. The correlation between the antigen test positivity and the RT-qPCR test incidence was significant for Round 1 (R-squared = 0.66 unweighted) but below the significance level 0.1 for Round 2 (see S1 Table for all parameter values).

## Discussion

The data from mass testing in Slovakia unveil spatial and temporal scales, patterns and trends that may be of general importance with consequences for public health policies. However, the scope of our results is limited to particular settings used in mass antigen testing in Slovakia and their global applicability requires caution. Particular attention should be paid to a difference between the observed test positivity and real infection prevalence in the population.

The most apparent feature in the data that we expect to generalize elsewhere is that the test positivity on the municipality level is exponentially distributed. We robustly observe it for all rounds regardless of whether testing was with or without mandatory mobility restrictions for untested individuals. Thus our analysis reveals a large degree of geographical heterogeneity contrary to claims in [15]. This heterogeneity is caused by the level of detail in the used dataset. While the authors of [15] considered solely the aggregated data on counties level we analyzed the full dataset resolved on the level of individual municipalities.

The exponential distribution of test positivity on the municipality level is also in agreement with the observed exponential spatial patterns. In Slovakia it is demonstrated by an exponential increase of the averaged test positivity in certain geographical directions on a long spatial scale comparable with the size of the country. However, the exponential distribution of test positivity is in contrast with the existing study [30] where the observed incidence distribution across different countries on a longer continental size scale followed a power-law distribution. We conjecture that the difference may be caused by significant biases for testing sample selection in regular (non-mass) testing that vary across considered countries.

Traditional epidemiological models predict approximately exponential temporal growth of a fraction of infected in a population in the early stages of epidemic [31]. A simple mechanism of a constant speed radial epidemic initiation and spreading from the localized sources is therefore consistent with spatially exponential trends observed in our analysis. This is in agreement with previous findings [32] that provide an explanation for the algebraic growth trends observed in epidemiological data globally [33, 34].

The characteristic spatial correlation length of test positivity is approximately 10 km. The level of spatial correlation of positivity on this scale in mass testing in Slovakia was significantly higher than previously observed elsewhere [20, 21] however, in other types of testing setups. Note that the same statistics for the total number of tests administered in Round 1 reveals a significantly different spatial scale 20–30 km (see S1 Fig). This indicates that the observed characteristic length scale of test positivity cannot be directly explained by the test volume that serves as a proxy for the total population.

Quantification of reduction of infected individuals by repeated mass testing is a central question in [7–9, 13, 15]. Our data analysis offers only the indirect information on this subject in quantification of the ratio of positivity in two consecutive mass testing rounds. Although we observe a systematic reduction of positivity in repeated testing on the county level in agreement with [15] we detect an increase of test positivity on average in repeated testing in low positivity municipalities (<0.25%). On the other hand, in municipalities with higher test positivity in the earlier round (>0.5%) the test positivity in the later round was reduced on average. The increasing trend in test positivity ratio between Rounds 1 and 2 with respect to the Round 1 positivity indicates that the effect of mass testing is spatially non-uniform and the repeated mass testing is most effective in the highest positivity regions in agreement with [7].

Also note that the vertical intercept of the weighted linear regression to the reduced dataset of the counties with small antigen test positivity (< 1%) (S1 Table, not shown in figure) provides a rough estimate of specificity of the antigen tests used (99.55–99.57%), since it represents the predicted (false) positivity at zero RT-qPCR incidence (0.43–0.45%). These values are in agreement with the manufacturer provided information and with additional validation studies (see Appendix A in S1 Text) and close to the minimum test specificity estimate in [29] that uses the same data set.

Participation in mass testing decreased significantly when testing in the later round was not connected with the stay-at-home order for untested individuals and the mass testing was only administered in municipalities with the high test positivity in the previous rounds. An average Round 3 positivity was systematically higher than in the earlier rounds indicating that the testing without mandated mobility restrictions for untested attracts a smaller testing sample but with a higher test positivity. Moreover, free voluntary testing offered only in certain locations in Round 3 attracted non-residents with higher positivity than residents despite the fact that testing was organized in the municipalities with the highest positivity in the previous rounds. It indicates that free voluntary testing may provide an efficient tool to identify antigen test positive individuals.

The results of the mass rapid antigen testing in Slovakia were also studied elsewhere [14–16, 29]. Unlike the other studies we primarily study spatial patterns and use the fine-resolved municipalities positivity data instead of coarsened county data used elsewhere. We also include the last round of mass testing that is omitted in other studies. We do not attempt to evaluate the success of the testing campaign as the long term effects of the campaign are not yet conclusive [13].

The data from mass antigen testing in Slovakia have significant limitations, see Appendix A in S1 Text for more details. No test validation was performed during the mass testing in Slovakia and test results were not confirmed by other laboratory diagnostics. Test parameters (sensitivity and specificity) may be sample-dependent as sensitivity depends on distribution of viral loads in the sample [35] and observed average viral loads may depend on current local epidemic dynamics [36]. Any eventual validation study should therefore be conducted under various epidemiological conditions.

Our results have implications for pandemic monitoring and for decisions on public health policies. The exponential distribution of positivity indicates that there are disproportionate numbers of municipalities with extreme test positivity values, only a few with relatively high positivity and many with relatively low positivity. Such information may aid efficient resource allocation during the pandemic and planning of mitigation strategies. Trends in geographical spreading, particularly if observed from areas in other countries, may serve as a basis for the decisions on border closing, mobility mitigation measures, and other interventions, as well as they may help to understand eventual sources of pandemic dynamics. The characteristic test positivity correlation length may contribute to an efficient localization of mitigation measures as it provides a quantitative estimate of the size of an area with the highest probability of correlation in the vicinity of known areas of high infection incidence.

## Supporting information

S1 Fig Moran’s global I statistics for the total number of administered tests. Spatial autocorrelation of the number of tests performed in Round 1 measured by the global Moran’s I statistics. See Fig 2 and Methods for more information. The code in MATLAB© is provided in the S1 Code and dataset.
(TIF)

S1 Text Test validation studies and survey of mitigation measures. Appendix A: Validation studies of the SD Biosensor Standard Q Covid Ag test. Appendix B: Survey of the additional mitigation measures imposed during mass antigen testing in Slovakia and specifics and limitations of tests and testing procedure.
(PDF)

S1 Table Parameters and confidence levels of the fitted models. Inference results for all models fitted in Fig 4, including other relevant models. Fits used are exponential y = C1*exp(-C2*x) in Fig 4a and linear y = C1*x+C2 in all other panels.
(PDF)

S1 Code and dataset Spreadsheet DataAG.xlsx contains all the used data. For the purpose of the numerical analysis the data are also stored in a MATLAB© table format, files DataAG.mat and Data_Counties.mat. The code is organized into four main files: figure1.m, figure2.m, figure3.m, and figure4.m. It was implemented in MATLAB© 2016b. MATLAB© curve fitting tool was also used only in figure4.m to compute weighted linear regressions.
(ZIP)
