# Supplementary figures and images for: Spatial scales, patterns, and positivity trends of SARS-CoV-2 pandemics in mass rapid antigen testing in Slovakia
Source: PLoS One. 2021 Aug 25;16(8):e0256669. doi: 10.1371/journal.pone.0256669 (PMC8386854; doi:10.1371/journal.pone.0256669)

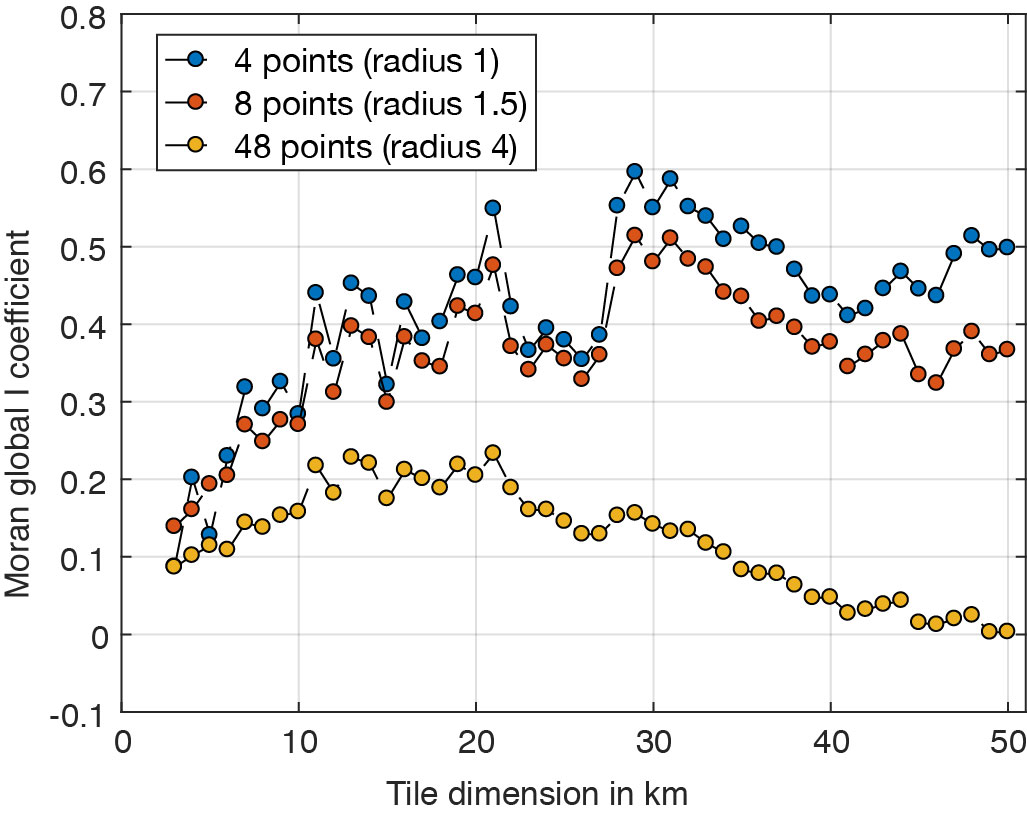

Supplement: S1 Fig — Spatial autocorrelation of the number of tests performed in Round 1 measured by the global Moran’s I statistics. See Fig 2 and Methods for more information. The code in MATLAB© is provided in the S1 Code and dataset. (TIF) [file pone.0256669.s001.tif]
