# Supplementary material for: Spatial scales, patterns, and positivity trends of SARS-CoV-2 pandemics in mass rapid antigen testing in Slovakia
Source: PLoS One. 2021 Aug 25;16(8):e0256669. doi: 10.1371/journal.pone.0256669 (PMC8386854; doi:10.1371/journal.pone.0256669)
Supplement: S1 Text — Appendix A: Validation studies of the SD Biosensor Standard Q Covid Ag test. Appendix B: Survey of the additional mitigation measures imposed during mass antigen testing in Slovakia and specifics and limitations of tests and testing procedure. (PDF) [file pone.0256669.s002.pdf]

Appendix A. Validation studies of the SD Biosensor Standard Q Covid Ag test

SD Biosensor Standard Q Covid Ag (SD Biosensor, Inc., Gyeonggi-do, Korea) [18, 19] is a rapid lateral flow chromatographic immunoassay for the qualitative detection of specific antigens to SARS-CoV-2 present in human nasopharynx. It provides results within 15-30 minutes that are evaluated by naked-eye.

Source	Country	Sample Size	RT-PCR positive % (n)	Sensitivity % (n)	Specificity % (n)
Berger A, et al. [2]	Switzerland	529	3.6% (191/529)	89.0% (170/191)	99.7% (1/338)
Linder AK, et al. [3]	Germany	289	13.5% (39/289)	79.5% (31/39)	99.6% (1/250)
Krueger LJ, et al. [4]	Germany/UK	1263	3.7% (47/1263)	76.6% (36/47)	99.3% (9/1216)
Cerutti F, et al. [5]	Italy	330	33.0% (109/330)	70.6% (77/109)	100.0% (0/221)
Sahar MK, et al. [6]	Egypt	100	80.0% (80/100)	68.7% (55/80)	95.0% (1/20)
FN Motol [7]*	Czechia	591	37.6% (222/591)	62.6% (139/222)	99.5% (2/369)
FIND [8]	Brazil	400	26.5% (106/400)	88.7% (/106)	97.6% (7/294)
Nalumansi A, et al. [9]	Uganda	262	34.3% (90/262)	70.0% (63/90)	92.4% (13/172)
Chaimayo C, et al. [10]	Thailand	454	13.2% (60/454)	98.3% (59/60)	98.7% (5/394)
Iglói Z, et al. [11]	Netherlands	970	19.2% (186/970)	84.9% (158/186)	99.6% (3/784)
Corman VM, et al. [12]	Germany	135	-	-	98.5% (133/135)
Salvagno GL, et al. [13]	Italy	321	46.4% (149/321)	72.5% (108/149)	99.4% (171/172)
Oh SM, et al. [14]	South Korea	118	33.9% (40/118)	17.5% (7/40)	100.0% (78/78)
Dinnes J, et al. [15] symptomatic	Meta analysis (64 studies)	-	-	88.0%	99.1%
Dinnes J, et al. [15] asymptomatic	Meta analysis (64 studies)	-	-	70.0%	99.1%
Van Honacker E, et al. [16]	Netherlands	4195	8.8% (369/4195)	54.2% (200/369)	99.7% (3814/3826)
Homza M, et al. [17]	Czechia	139	30.2% (42/139)	61.9% (26/42)	99.0% (96/97)
Average				72.1%	98.6%
Median				71.6%	99.3%

Table A: Validation studies of the SD Biosensor Standard Q Covid Ag (SD Biosensor, Inc., Gyeonggi-do, Korea) test [18] also distributed by Roche [19]. *Test #2 in the validation study is SD Biosensor Standard Q Covid Ag test. The sample tested in the mass testing in Slovakia may significantly statistically differ from the validation studies samples.

Appendix B. Additional mitigation measures imposed during mass antigen testing in Slovakia and specifics and limitations of tests and testing procedure

All individuals with positive test results were ordered to isolate for 10 days with their whole households. All persons with negative test results in the pilot, and in Rounds 1-2 of mass testing were issued an official certificate that allowed them to avoid some of the strict measures enforced during the following 7-day period (or 14-day in counties without the second testing round). During the period all persons without a confirmation of a negative antigen test or a recent real time quantitative negative polymerase chain reaction (RT-qPCR) test were subject to a mandated isolation. They were allowed to leave their household only during the night (01:00-05:00 am), or to leave their household to take a RT-qPCR or an antigen test, to visit the nearest grocery store or a pharmacy, or to get medical care. They were also allowed to provide assistance and personal care for their close persons or livestock, to walk pets up to 100 meters away from their household, and to attend a funeral. Those with negative tests were in addition allowed to travel to work, accompany their children to school, to visit post offices, insurance companies, drycleaning, car repair shops, and petrol stations. They were allowed to spend time in nature within the counties of their residence outside of urban areas.

More details on extent and timing of mitigation measures during testing and testing procedures can be found in [20].

Tests were administered at temporary set testing stations. They were staffed by volunteers, each testing team included among others two medical professionals who collected samples and evaluated tests and one member of the army forces who coordinated testing locally and reported the data to the army regional headquarters for data collection. Testing stations were located both indoors and outdoors, a minority of testing stations were drive-through.

According to the test package leaflet "the test is for administration by healthcare workers and labs only, as an aid to early diagnosis of SARS-CoV-2 infection in patients with clinical symptoms with SARS-CoV-2 infection. It provides only an initial screening test result... The result of this test should not be the sole basis for the diagnosis; confirmatory testing is required" [18]. During the mass testing in Slovakia test results were not confirmed by other laboratory diagnostics and most of the tested individuals were asymptomatic.

The manufacturer also recommends that the tests are at room temperature prior to sample collection. However, during the mass testing, many testing stations were for epidemiological reasons located in outdoor settings and the temperature at many testing stations was under the CDC recommended room temperature (15-30°C) before use [21]. The sample collection was performed by volunteer medical personnel that did not receive any specific training on how to perform the pharangonasal swabs. Also no specific training was provided for an evaluation of the tests. Also 20 Euro risk compensation was paid to staff collecting the sample for each positive test, however, the test was evaluated by a different member of the testing team that did not receive any risk compensation.

The test data do not contain information on individuals tested outside of their area of the residence in Rounds 1 and 2 and they may also contain duplicities caused by individuals tested repeatedly within one round. The relation between test positivity in individual rounds is also influenced by the local speed of epidemic growth. The local effective reproduction number may significantly contribute to the relative change of the test positivity between two rounds. This factor prohibits a simple interpretation of the reduction of the infected in the population by the mass testing effort.

References

1 Blasius B. Power-law distribution in the number of confirmed COVID-19 cases. *Chaos* 2020;30(9):093123.
doi: https://aip.scitation.org/doi/10.1063/5.0013031

2 Berger A, Nsoga MTN, Perez-Rodriguez FJ, et al. (2021) Diagnostic accuracy of two commercial SARS-CoV-2 antigen-detecting rapid tests at the point of care in community-based testing centers. PLoS ONE 16(3): e0248921.
doi: https://doi.org/10.1371/journal.pone.0248921

3 Lindner AK, Nikolai O, Kausch F, et al. Head-to-head comparison of SARS-CoV-2 antigen-detecting rapid test with self-collected nasal swab *versus* professional-collected nasopharyngeal swab. *European Respiratory Journal 2021 57: 2003961.*
doi: https://doi.org/10.1183/13993003.03961-2020

4 Krüger LJ, Gaeddert M, Köppel L, et al. Evaluation of the accuracy, ease of use and limit of detection of novel, rapid, antigen-detecting point-of-care diagnostics for SARS-CoV-2. MedRxiv 20203836 [Preprint]. October 04, 2020 (Date accessed: May 2021)
doi: https://doi.org/10.1101/2020.10.01.20203836

5 Cerutti F, Burdino E, Milia MG, et al. Urgent need of rapid tests for SARS CoV-2 antigen detection: Evaluation of the SD-Biosensor antigen test for SARS-CoV-2. *J Clin Virol* 2020;132**:**104654.
doi: https://doi.org/10.1016/j.jcv.2020.104654

6 Khairat SM, Guindy NEL, Motaleb MSEA, et al. Evaluation of Two Rapid Antigen Tests for Detection of SARS-CoV-2 Virus, *International Journal of Microbiology and Biotechnology* 2020;5(3):131-134.
doi: https://doi.org/10.11648/j.ijmb.20200503.18

7 Faculty Hospital Motol, Prague, Czech Republic. Validation study of rapid antigen tests in Czech Republic. 2020 [cited 2021 31 May]. Available from:
https://www.fnmotol.cz/_sys_/FileStorage/download/3/2854/priloha_vysledky-srovnavaci-studie-antigen-vs-pcr.pdf

8 FIND Evaluation of SD Biosensor, Inc. STANDARD Q COVID-19 Ag Test External Report Version 1.0, 18 September 2020 [cited 2021 31 May]. Available from: https://www.finddx.org/wp-content/uploads/2020/09/SDQ-Ag-Public-Report_20200918.pdf

9 Nalumansi A, Lutalo T, Kayiwa J, et al. Field Evaluation of the Performance of a SARS-CoV-2 Antigen Rapid Diagnostic Test in Uganda using Nasopharyngeal Samples, *International Journal of Infectious Diseases* 2021; 104: 282-286.
doi: https://doi.org/10.1016/j.ijid.2020.10.073

10 Chaimayo C, Kaewnaphan B, Tanlieng N, et al. Rapid SARS-CoV-2 antigen detection assay in comparison with real-time RT-PCR assay for laboratory diagnosis of COVID-19 in Thailand. *Virol J* 2020;17:177.
doi: https://doi.org/10.1186/s12985-020-01452-5

11 Iglὸi Z, Velzing J, van Beek J et al. Clinical evaluation of the Roche/SD Biosensor rapid antigen test with symptomatic, non-hospitalized patients in a municipal health service drive-through testing site. MedRxiv: 20234104 [Preprint]. 2020 [cited 2021 May 31]. Available from: https://doi.org/10.1101/2020.11.18.20234104

12 Corman VM, Haage VC, Bleicket T, et al. Comparison of seven commercial SARS-CoV-2 rapid Point-of-Care Antigen tests. Lancet Microbe 2021;Forthcoming.
doi: https://doi.org/10.1016/S2666-5247(21)00056-2

13 Salvagno GL, Gianfilippi G, Bragantini D, Henry BM and Lippi G. Clinical assessment of the Roche SARS-CoV-2 rapid antigen test. Diagnosis 2021;pp. 000010151520200154, Forthcoming.
doi: https://doi.org/10.1515/dx-2020-0154

14 Oh SM, Jeong H, Chang E, et al. Clinical Application of the Standard Q COVID-19 Ag Test for the Detection of SARS-CoV-2 Infection. J Korean Med Sci. 2021 Apr; 36(14):e101.
doi: https://doi.org/10.3346/jkms.2021.36.e101

15 Dinnes J, Deeks JJ, Berhane S, et al. Rapid, point-of-care antigen and molecular-based tests for diagnosis of SARS-CoV-2 infection. Cochrane Database of Systematic Reviews 2021;3:CD013705.
doi: https://doi.org/10.1002/14651858.CD013705.pub2

16 Van Honacker E, Van Vaerenbergh K, Boel A, et al. Comparison of five SARS-CoV-2 rapid antigen tests in a hospital setting and performance of one antigen assay in routine practice. A useful tool to guide isolation precautions? Journal of Hospital Infection. 2021; Forthcoming.
doi: https://doi.org/10.1016/j.jhin.2021.03.021

17 Homza M, Zelena H, Janosek J, et al. Five Antigen Tests for SARS-CoV-2: Virus Viability Matters. Viruses 2021;13:684.
doi: https://doi.org/10.3390/v13040684

18 SD Biosensor COVID-19 Ag STANDARD™ Q COVID-19 Ag Test. Package leaflet. 2020 [cited 2021 31 May]. Available from: http://sdbiosensor.com/xe/product/7672

19 Roche to launch SARS-CoV-2 Rapid Antigen Test in countries accepting CE mark, allowing fast triage decisions at point of care. Media release. 2020 [cited 2021 31 May]. Available from: https://www.roche.com/media/releases/med-cor-2020-09-01b.htm

20 Pavelka M, Van-Zandvoort K, Abbott, S, et al. The impact of population-wide rapid antigen test testing on SARS-CoV-2 prevalence in Slovakia. Science 2021;372: 635–641.
doi: https://doi.org/10.1126/science.abf9648

21 Centers for Diseases Control and Prevention. Interim Guidance for Antigen Testing for SARS-CoV-2. 2020 Dec 16 [Cited 2021 May 31]. Available from: https://www.cdc.gov/coronavirus/2019-ncov/lab/resources/antigen-tests-guidelines.html
