# Supplementary material for: Spatial scales, patterns, and positivity trends of SARS-CoV-2 pandemics in mass rapid antigen testing in Slovakia
Source: PLoS One. 2021 Aug 25;16(8):e0256669. doi: 10.1371/journal.pone.0256669 (PMC8386854; doi:10.1371/journal.pone.0256669)
Supplement: S1 Table — Inference results for all models fitted in Fig 4, including other relevant models. Fits used are exponential y = C1*exp(-C2*x) in Fig 4a and linear y = C1*x+C2 in all other panels. (PDF) [file pone.0256669.s003.pdf]

Parameters and confidence levels of the fitted models

**Fitted data**	**C1 (95% CI)**	**C2 (95% CI)**	**R-squared**	**n**
Fig. 4a, R1, municipalities	706.17 (692.61,720.00)***	0.88 (0.86,0.91)***	0.9975	2681
Fig. 4a, R2, municipalities	293.94 (263.49, 327.90)***	1.52 (1.29,1.76)***	0.9185	1580
Fig. 4a, R3, municipalities	22.23 (19.23, 25.71)***	0.43 (0.35,0.51)***	0.8517	447
Fig. 4b, R12, municipalities	0.17 (0.15-0.20)***	0.36 (0.32,0.41)***	0.10	1580
Fig. 4b, R23, municipalities	0.35 (0.05-0.65)*	0.79 (0.26,1.33)**	0.02	314
Fig. 4b, R12, municipalities weighted	0.24 (0.22-0.28)***	0.28 (0.25,0.32)***	0.23	1580
Fig. 4b, R23, municipalities weighted	0.29 (-0.05,0.47)^{ns}	1.18 (0.78,1.58)***	0.008	314
Fig. 4c, R1, counties weighted	2.46 (1.82-3.10)***	0.28 (0.04-0.51)*	0.46	72
Fig. 4c, R2, counties weighted	0.34 (-0.07-0.74)^{ns}	0.50 (0.32-0.68)***	0.06	45
Fig. 4c, R1, counties weighted Ag<1%	0.74 (0.00,1.48)*	0.43 (0.27,0.59)***	0.11	34
Fig. 4c, R2, counties weighted Ag<1%	0.39 (0.09,0.68)*	0.45 (0.31,0.58)***	0.15	43
Fig. 4d, R12, counties	0.38 (0.30-0.46)***	0.07 (-0.07,0.21)^{ns}	0.66	45
Fig. 4d, R12, counties weighted	0.34 (0.27-0.42)***	0.12 (0.00-0.25)*	0.65	45

S1 Table: Inference results for all models fitted in Fig. 4, including other relevant models. Fits used are an exponential y=C1*exp(-C2*x) in Fig. 4a and a linear y=C1*x+C2 in all other panels.
