# Supplementary material for: Spatial scales, patterns, and positivity trends of SARS-CoV-2 pandemics in mass rapid antigen testing in Slovakia
Source: PLoS One. 2021 Aug 25;16(8):e0256669. doi: 10.1371/journal.pone.0256669 (PMC8386854; doi:10.1371/journal.pone.0256669)
Supplement: S1 Code and dataset — For the purpose of the numerical analysis the data are also stored in a MATLAB© table format, files DataAG.mat and Data_Counties.mat. The code is organized into four main files: figure1.m, figure2.m, figure3.m, and figure4.m. It was implemented in MATLAB© 2016b. MATLAB© curve fitting tool was also used only in figure4.m to compute weighted linear regressions. (ZIP) [file pone.0256669.s004.zip › supplement_S2/readme.rtf]

All data are stored in the spreadsheet DataAG.xlsx, but for the purpose of the analysis we stored them also in a Matlab table format in files DataAG.mat and Data_Counties.mat. Our code was written in Matlab 2016b with the use of a curve fitting tool. This tool is used only in figure4.m to compute weighted linear regressions.Each of the m-files figure1.m, figure2.m, figure3.m, and figure4.m generates all plots in the manuscript (after setting the appropriate options in the code). For visualisation purposes we used two functions, which are available for download on File Exchange:Ian Stevenson (2020). beeswarm (https://github.com/ihstevenson/beeswarm), GitHub. Retrieved December 13, 2020. Rob Campbell (2020). raacampbell/shadedErrorBar (https://github.com/raacampbell/shadedErrorBar), GitHub. Retrieved December 13, 2020. 
